# Hematological and Hemodynamic Responses to Acute and Short-Term Creatine Nitrate Supplementation

**DOI:** 10.3390/nu9121359

**Published:** 2017-12-15

**Authors:** Ryan L. Dalton, Ryan J. Sowinski, Tyler J. Grubic, Patrick B. Collins, Adriana M. Coletta, Aimee G. Reyes, Brittany Sanchez, Majid Koozehchian, Yanghoon P. Jung, Christopher Rasmussen, Mike Greenwood, Peter S. Murano, Conrad P. Earnest, Richard B. Kreider

**Affiliations:** 1 Exercise and Sport Nutrition Lab, Human Clinical Research Facility, Texas A & M University, College Station, TX 77843-4253, USA; ryanldalton@exchange.tamu.edu (R.L.D.); ryansowinski6@gmail.com (R.J.S.); tylergrubic@tamu.edu (T.J.G.); blaise_collins@tamu.edu (P.B.C.); AMColetta@mdanderson.org (A.M.C.); agreyes1@exchange.tamu.edu (A.G.R.); bksanchez@csub.edu (B.S.); mkoozehchian@jsu.edu (M.K.); yp.jung@cj.net (Y.P.J.); crasmussen@tamu.edu (C.R.); mgreenwood26@tamu.edu (M.G.); conradearnest@exchange.tamu.edu (C.P.E.); 2 Institute for Obesity and Program Evaluation, Texas A & M University, College Station, TX 77843, USA; psmurano@tamu.edu; 3 Clinical Science Division, Nutrabolt, 3891 S. Traditions Drive, Bryan, TX 77807, USA

**Keywords:** creatine, nitrate, safety, dietary supplement, ergogenic aid

## Abstract

In a double-blind, crossover, randomized and placebo-controlled trial; 28 men and women ingested a placebo (PLA), 3 g of creatine nitrate (CNL), and 6 g of creatine nitrate (CNH) for 6 days. Participants repeated the experiment with the alternate supplements after a 7-day washout. Hemodynamic responses to a postural challenge, fasting blood samples, and bench press, leg press, and cycling time trial performance and recovery were assessed. Data were analyzed by univariate, multivariate, and repeated measures general linear models (GLM). No significant differences were found among treatments for hemodynamic responses, clinical blood markers or self-reported side effects. After 5 days of supplementation, one repetition maximum (1RM) bench press improved significantly for CNH (mean change, 95% CI; 6.1 [3.5, 8.7] kg) but not PLA (0.7 [−1.6, 3.0] kg or CNL (2.0 [−0.9, 4.9] kg, CNH, *p* = 0.01). CNH participants also tended to experience an attenuated loss in 1RM strength during the recovery performance tests following supplementation on day 5 (PLA: −9.3 [−13.5, −5.0], CNL: −9.3 [−13.5, −5.1], CNH: −3.9 [−6.6, −1.2] kg, *p* = 0.07). After 5 days, pre-supplementation 1RM leg press values increased significantly, only with CNH (24.7 [8.8, 40.6] kg, but not PLA (13.9 [−15.7, 43.5] or CNL (14.6 [−0.5, 29.7]). Further, post-supplementation 1RM leg press recovery did not decrease significantly for CNH (−13.3 [−31.9, 5.3], but did for PLA (−30.5 [−53.4, −7.7] and CNL (−29.0 [−49.5, −8.4]). CNL treatment promoted an increase in bench press repetitions at 70% of 1RM during recovery on day 5 (PLA: 0.4 [−0.8, 1.6], CNL: 0.9 [0.35, 1.5], CNH: 0.5 [−0.2, 0.3], *p* = 0.56), greater leg press endurance prior to supplementation on day 5 (PLA: −0.2 [−1.6, 1.2], CNL: 0.9 [0.2, 1.6], CNH: 0.2 [−0.5, 0.9], *p* = 0.25) and greater leg press endurance during recovery on day 5 (PLA: −0.03 [−1.2, 1.1], CNL: 1.1 [0.3, 1.9], CNH: 0.4 [−0.4, 1.2], *p* = 0.23). Cycling time trial performance (4 km) was not affected. Results indicate that creatine nitrate supplementation, up to a 6 g dose, for 6 days, appears to be safe and provide some ergogenic benefit.

## 1. Introduction

Creatine has proven to be a safe and effective nutritional ergogenic aid for improving high intensity exercise performance [1,2,3,4,5]. The primary form of creatine studied to date is creatine monohydrate [1,3]. Short-term creatine monohydrate supplementation (i.e., 0.3 g/kg/day for 3–7 days) and/or long-term creatine supplementation (e.g., 0.03 g/kg/day) has been reported to increase muscle phosphocreatine levels by 10–40%, improve high-intensity exercise performance, and enhance training adaptations, leading to an increase in muscle mass [1,6,7,8,9,10,11,12,13,14,15]. More recently, a number of clinical applications of creatine supplementation have been studied involving neurodegenerative diseases (e.g., muscular dystrophy, Parkinson’s, Huntington’s disease), diabetes, osteoarthritis, fibromyalgia, aging, brain and heart ischemia, adolescent depression, and pregnancy [1]. Collectively, these studies provide a large body of evidence that creatine can not only improve exercise performance, but can play a role in preventing and/or reducing the severity of injury, enhancing rehabilitation from injuries, and helping athletes tolerate heavy training loads [1,16]. For this reason, creatine is one of the most common nutrients found in dietary supplements marketed to active individuals and it is increasingly being recommended as a dietary strategy to support general health as one ages [1,3,16].

Over the years, a number of different forms of creatine have been introduced into the market, each purported to promoted greater bioavailability and/or efficacy [3]. Our group has evaluated the efficacy and safety of a number of newer forms of creatine, including serum creatine [17], effervescent creatine [18], creatine ethyl ester [19], and a buffered form of creatine [20]. These studies have shown that while these forms of creatine may offer some benefits, they do not appear to promote greater adaptations than creatine monohydrate [3]. Additionally, we have investigated whether co-ingestion of creatine with carbohydrate [18], protein [6], carbohydrate and protein weight gain supplements [8,11], D-pinitol [21], fenugreek extract [22], beta alanine [23], and Russian Tarragon [24] enhance the bioavailability and/or benefits than creatine supplementation alone. These studies have generally indicated that co-ingesting creatine with other nutrients that have been shown to enhance creatine retention, exercise capacity, and/or training adaptations can provide synergistic and/or additive benefits.

More recently, nitrate supplementation, primarily in the form of beetroot juice or inorganic nitrates, has been reported to improve exercise capacity [25,26,27,28,29,30,31,32,33,34,35,36,37,38]. The ergogenic dose of nitrates is about 300 mg, ingested 1 to 2 h prior to exercise or for several days prior to performance [30,39]. For example, Lansley and colleagues [27] reported that acute dietary nitrate supplementation increases 4- and 16.1-km cycling time trial performance. Clifford and coworkers [40] reported that beetroot juice ingestion reduced the decrement in jumping performance and reactive strength index following repeated sprint training. Mosher et al. [41] reported that nitrate supplementation (400 mg) increased the number of repetitions performed to failure and total work performed during resistance training. Finally, beetroot ingestion has been reported to enhance repetitive sprint performance [37,38]. For this reason, a number of dietary supplements are now available containing beetroot and/or inorganic nitrates. Additionally, it has been recommended that individuals consume a diet high in nitrates (e.g., celery, cress, lettuce, red beetroot, spinach, rucola) with a goal of ingesting 1200 mg/day or more of nitrates as a means of managing hypertension and/or reducing risk to cardiovascular disease [42,43].

Creatine nitrate (i.e., C_4_H_10_N_4_O_5_) was introduced to the market about six years ago. It is generally formed by combining nitric acid and creatine with water until crystallized into creatine dinitrate or creatine trinitrate [44]. Alternatively, it can be formed using nitrous acid instead of nitric acid and yielding creatine nitrate [44]. The rationale in combining creatine with nitrate was to enhance vasodilation and thereby theoretically enhance creatine absorption and/or efficacy [44]. While creatine and nitrate have been studied independently, less is known about the effects of creatine nitrate supplementation on exercise performance and/or recovery and some have expressed concerns over potential health risks [45,46,47,48,49,50]. Joy and colleagues [51] reported that ingestion of 1 or 2 g/day of creatine nitrate for 28 days appeared to be safe in healthy individuals. Our group recently reported that acute ingestion of 1.5 g and 3 g of creatine nitrate did not increase muscle creatine content as much as creatine monohydrate or affect acute heart rate, blood pressure, or hematological responses [52]. Additionally, 28 days of creatine nitrate supplementation during training (i.e., 6 or 12 g/day for 7 days and 1.5 g or 3 g/day for 21 days) did not increase muscle creatine levels to the same degree as creatine monohydrate. However, some performance advantages were seen in comparison to creatine monohydrate, apparently due to increased nitrate availability. We also evaluated the acute (one dose) and chronic (28 days) effects of ingesting a pre-workout supplement containing 2 g per serving of creatine nitrate as part of a pre-workout supplement [53,54]. These studies did not identify any significant negative side effects from acute or chronic creatine nitrate supplementation. However, more research is needed, particularly at the higher doses that some athletes may take, to examine safety and efficacy.

The purpose of this study was to examine the acute and short-term effects (0, 1, 5 and 6 days) of ingesting 3 g and 6 g doses of creatine nitrate on hemodynamic responses to a postural challenge, prior to and following, exercise, exercise performance, and recovery. The primary outcome was an assessment of safety, as determined by evaluating hemodynamic responses to a postural challenge, prior to and following, intense exercise, fasting hematology, and self-reported symptoms and side effects. The secondary outcome was an assessment of performance, as determined by examining upper and lower body one repetition maximum (1RM), muscular endurance, and 4 km cycling time trial performance. We hypothesized that acute and short-term creatine nitrate would enhance performance and/or recovery, while posing no adverse side effects.

## 2. Methods

### 2.1. Experimental Design

**Figure 1:**
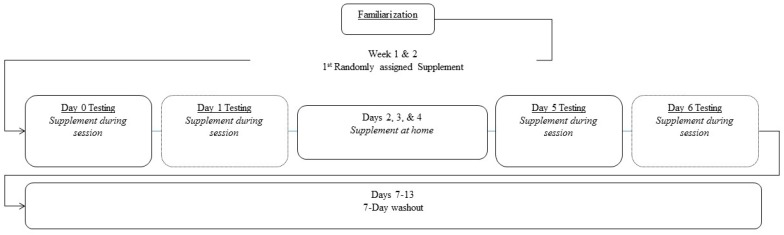
Overview of study design.

**Figure 2:**
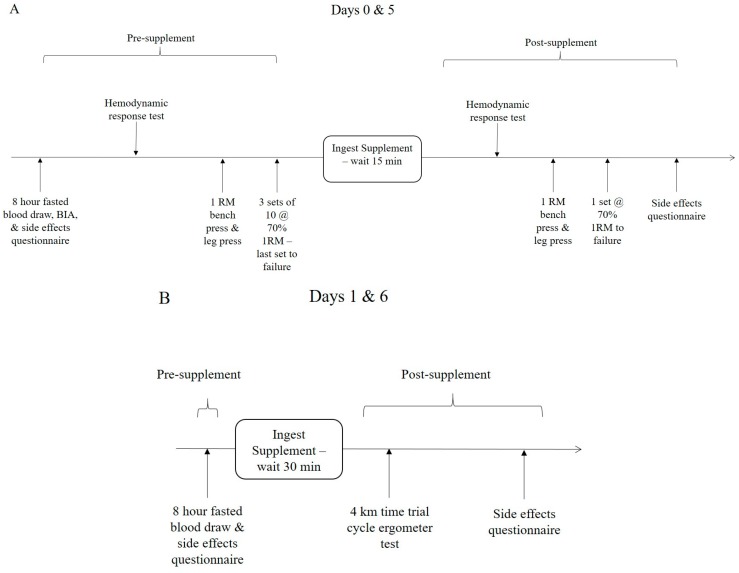
Testing sequence timeline. (**A**) Presents the testing sequence on days 0 and 5, while (**B**) shows the testing sequence on days 1 and 6 of supplementation.

Prior to starting the study, approval was obtained from the Texas A & M University Institutional Review Board (IRB2015-0684F). This study is also registered with clinicatrials.gov (#NCT03039829). This study was conducted at a university-based research setting in a double-blind, randomized, counter-balanced, crossover, and repeated measures manner, after obtaining informed consent from each participant. The independent variable was nutritional supplementation. Figure 1 shows the order of tests performed for each experiment, while Figure 2 presents the order of testing on each testing day. The study design allowed for the assessment of acute and short-term responses to creatine nitrate supplementation on performance and recovery from resistance exercise. The following describes the general methods employed.

### 2.2. Participants

**Figure 3:**
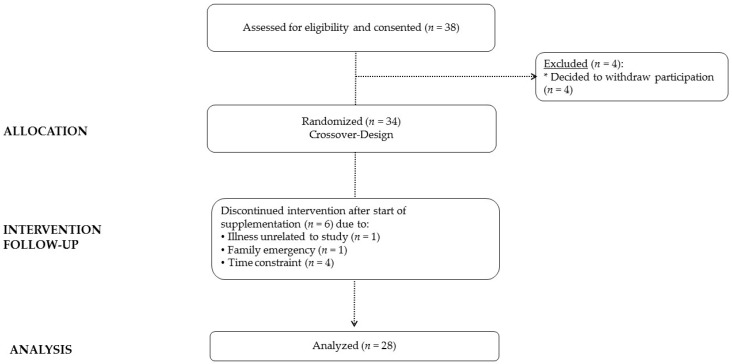
CONSORT diagram.

Figure 3 presents a CONSORT diagram. Apparently healthy and recreationally active men and women, between the ages 18–40 years, were recruited to participate in this study. Individuals who expressed interest from email or study advertisements were interviewed to determine if they met initial screening eligibility to participate in this study. Those who met initial screening qualifications, were invited to attend a familiarization session in which they received written and verbal explanations of the study design, and testing procedures, and were able to review the informed consent document. Interested individuals signed the informed consent and then completed personal and medical histories and had height, weight, resting blood pressure, and heart rate measured. A registered nurse reviewed medical history forms and physical examination measurements to determine eligibility to participate. Inclusion criteria required that each participant have at least 6 months of resistance training immediately prior to entering the study, inclusive of performing bench press and leg press or squats. Participants were excluded from participation if they had a history of treatment for metabolic disease (i.e., diabetes), hypertension, hypotension, thyroid disease, arrhythmias, and/or cardiovascular disease; they were currently using any prescription medication (birth control was allowed); they were a pregnant or lactating female or planned to become pregnant within the next month; they had a history of smoking; they drank excessively (12 drinks per week); or they had a recent history of creatine or nitrate supplementation within eight weeks of the start of supplementation. Participants were 21.6 ± 3.7 years of age, 172.1 ± 8.2 cm tall, and weighed 73.4 ± 10.9 kg.

### 2.3. Familiarization

Participants who met eligibility criteria and were cleared to participate in the study underwent a familiarization session in which Dual-Energy X-ray Absorptiometry determined body composition and bioelectrical impedance (BIA) determined body water measurements were obtained. Additionally, participants completed 1RM muscular strength and 70% of 1RM muscular endurance tests on the bench press and leg press. This was followed by warming up on a cycle ergometer and performing a 4 km cycle ergometer time trial. These tests served to familiarize the participants with the testing procedures and served as baseline performance comparators. After exercise testing was completed, participants scheduled their lab visits (4 testing sessions per week of each treatment experiment followed by a 7-day washout period between experiments).

### 2.4. Supplementation Protocol

Participants were assigned in a randomized, counter balanced, double-blind, and cross-over manner, to one of three supplement treatments for each testing week, while following their normal diet. The supplements consisted of: (1.) 6.0 g of a dextrose placebo (PLA); 3.0 g of creatine nitrate (2 g creatine; 1 g nitrate, CNL) with 3.0 g of dextrose; or, (2.) 6.0 g of creatine nitrate (4 g creatine; 2 g nitrate, CNH). Nutrabolt (Bryan, TX, USA) provided all of the supplements for this study. Supplements were prepared and packaged by Thermo-life International (Phoenix, AZ, USA). All supplements were provided in identical clear plastic packets, with the only differentiating characteristic being the letter A, B, or C printed on the label of each packet. All supplements were indistinguishable from each other, based on taste, texture, and appearance. Supplements were administered in a double-blinded manner. Participants were instructed to mix the entire contents of the packet with 16 ounces of water. Compliance was monitored at each testing session by administering the supplements and observing ingestion as well as from side effect questionnaires.

During each 6-day experiment, supplements were administered after completing the first bout of exercise on day 0. On day 1, the second supplement dose was consumed after donating blood in a fasted state. Participants were given 3 packets of supplements after completing testing on day 1 and were instructed to ingest one packet, mixed with water, each day for the next three days (Days 2, 3, and 4). On day 5, participants ingested the sixth dose of their assigned supplement, immediately following the first round of exercise. On day 6, participants consumed their seventh and final dose of their respective supplement, immediately after donating a blood sample in a fasted state. Thus, participants consumed 7 doses of their assigned supplement (1 per day) over a 6-day period, followed by a 7-day washout period where no supplement was consumed, nor testing performed. After this, the supplementation and testing schedule was repeated with the participants’ next assigned supplement. This schedule was repeated a total of 3 times (Figure 1), in a counterbalanced and double-blind manner.

### 2.5. Testing Sequence

Participants arrived at the laboratory on days 0, 1, 5, and 6 during each treatment period. Participants were instructed to refrain from exercise, alcohol, and non-steroidal anti-inflammatory drugs (NSAID) consumption for 48 h prior to each testing session. All fasting blood samples were obtained following an 8 h fast, primarily between the hours of 6:00–9:00 a.m. On days 0 and 5 (Figure 2A), participants donated a venous blood sample and completed a pre-exercise side effects questionnaire. Participants were then weighed and BIA total body water measurements were obtained. Participants then performed a pre-supplementation hemodynamic postural challenge test, using a tilt table, which consisted of obtaining heart rate and blood pressure measurements in supine and vertical positions, prior to and following a postural challenge. Subjects then performed 1RM tests on the bench press and leg press, followed by performing 3 sets of 10 repetitions at 70% of 1RM, with the last set to failure. Participants then ingested their assigned supplement and waited 15 min before repeating the hemodynamic reactivity test. Approximately 30 min after ingestion of the supplement, participants repeated 1RM tests on the bench press and leg press. This was followed by performing 1 set to failure at 70% of 1RM on the bench press and leg press. The rationale for this approach was to determine whether ingestion of the supplements would influence exercise capacity after exhaustive exercise and toward the end of a training session. Participants then completed a side effects questionnaire.

On days 1 and 6 (Figure 2B), participants reported to the lab at approximately the same time of day following an 8 h fast and donated a fasting blood sample. This was followed by completing a side effects questionnaire. The subject then ingested one dose of their assigned supplement. Approximately 30 min after ingesting the supplement, the participant warmed up and performed a 4 km cycling time trial on an electronically-braked ergometer. Participants were encouraged to complete the 4 km distance as quickly as possible. After completing the time trial, participants completed a post exercise side effects questionnaire. Participants observed a 7-day washout period before repeating the experiment while consuming the alternate treatments.

## 3. Procedures

### 3.1. Anthropometry & Body Composition

Standardized anthropological testing included assessments for body mass and height, on a Healthometer Professional 500KL (Pelstar LLC, Alsip, IL, USA) self-calibrating digital scale, with an accuracy of ±0.02 kg. Total body water was determined under standardized conditions, using an ImpediMed DF50 BIA analyzer (ImpediMed, San Diego, CA, USA). Whole body bone density and body composition measures (excluding cranium) were determined with a Hologic Discovery W DEXA (Hologic Inc., Waltham, MA, USA) equipped with APEX Software (APEX Corporation Software, Pittsburg, PA, USA) by using standardized procedures [55,56]. On the day of each test, the equipment was calibrated following the manufacturer’s guidelines.

### 3.2. Hemodynamic Challenge Assessment

Participants were placed on a standard tilt table in a supine position (IRONMAN Gravity 4000 Inversion Table; Paradigm Health & Wellness, Inc., City of Industry, CA, USA). After 15 min of motionless rest, heart rate was taken at the radial artery and systolic and diastolic blood pressure was measured by listening for Korotkoff sounds from the brachial artery at the antecubital area of the elbow using standard stethoscopes and sphygmomanometers. Next, the tilt table was adjusted to vertical, where the participant rested for 2 min and the metrics were re-assessed. Mean arterial pressure was calculated as 1/3 systolic blood pressure plus 2/3 diastolic blood pressure [57]. Pulse pressure was calculated as the difference between systolic and diastolic blood pressure [58]. Rate pressure product was calculated as heart rate multiplied by systolic blood pressure [59].

### 3.3. Blood Collection Procedures

Participants provided an 8 h fasted blood sample via venipuncture of an antecubital vein in the forearm, in accordance with standard phlebotomy procedures. Approximately 10 mL of whole blood was collected at the beginning of each testing day, in one 3.5 mL BD Vacutainer^®^ K2 EDTA tube (Becton, Dickinson and Company, Franklin Lakes, NJ, USA) and one 8.5 mL BD Vacutainer^®^ serum separation tube (Becton, Dickinson and Company, Franklin Lakes, NJ, USA). Both tubes sat at room temperature for 15 min, then the 8.5 mL serum separation tube was centrifuged at 3500 rpm for 10 min using a 4 °C refrigerated bench top ThermoScientific Heraeus MegaFuge 40R Centrifuge (Thermo Electron North America LLC, West Palm Beach, FL, USA). Both tubes were stored at 4 °C for 3 to 4 h, prior to analysis or storage. Serum was stored at −80 °C in polypropylene microcentrifuge tubes for later analysis.

### 3.4. Blood Chemistry Analysis

A complete blood count with platelet differential (hemoglobin, hematocrit, red blood cell counts, mean corpuscular volume (MCV), mean corpuscle hemoglobin (MCH), mean corpuscular hemoglobin concentration (MCHC), red blood cell distribution width (RDW), white blood cell counts, lymphocytes, granulocytes, and mid-range absolute count (MID)) was measured using a Abbott Cell Dyn 1800 (Abbott Laboratories, Abbott Park, IL, USA) automated hematology analyzer. The internal quality control for Abbott Cell Dyn 1800 was performed using three levels of control fluids purchased from manufacturer to calibrate acceptable SD and CV values for all whole blood cell parameters. Test-to-test reliability assessment of assays evaluated in the study yielded mean CVs of <6.3% with *r* values > 0.9.

Serum blood samples were analyzed for the following: glucose, alkaline phosphatase (ALP), aspartate transaminase (AST), alanine transaminase (ALT), creatinine, blood urea nitrogen (BUN), creatine kinase (CK), lactate dehydrogenase (LDH), glucose, total cholesterol, high density lipoprotein (HDL), low density lipoprotein (LDL), and triglycerides (TG), using a Cobas c111 (Roche Diagnostics, Basel, Switzerland) automated clinical chemistry analyzer. The analyzer was calibrated daily, as per the manufacturer’s guidelines and has been shown to be valid and reliable in previously published reports [33]. Internal quality control was performed using two levels of control fluids purchased from the manufacturer to calibrate acceptable standard deviation (SD) and coefficient of variation (C_v_) values for all assays. Samples were re-run if the values observed were outside control values and/or clinical norms, according to standard procedures. A prior analysis in our lab has yielded test-to-test reliability of a range of CVs, from 0.4 to 2.4% for low control samples and 0.6–1.9% for high controls. Precision has been found between 0.8 and 2.4% for low controls and 0.5–1.7% for high controls.

### 3.5. Side Effects

Side effects questionnaires were used to document how well participants tolerated each treatment as well as to monitor compliance to the supplementation protocol. Participants were asked to rank the frequency and severity of dizziness, headache, tachycardia, heart skipping or palpitations, shortness of breath, nervousness, blurred vision, and unusual or adverse effects. Participants were requested to rank their perceived symptoms with 0 (none), 1 (minimal: 1–2/week), 2 (slight: 3–4/week), 3 (occasional: 5–6/week), 4 (frequent: 7–8/week), or 5 (severe: 9 or more/week).

### 3.6. Muscular Strength and Endurance Assessment

Bench press tests were performed using a standard isotonic Olympic bench press (Nebula Fitness, Versailles, OH, USA), while leg press was determined using a hip/leg sled (Nebula Fitness, Versailles, OH, USA), using standard procedures [60]. Participants performed three warm up sets, prior to performing 1RM attempts (i.e., one set of 10 at 50%, one set of 5 at 70%, and one set of 3 at 90% of anticipated 1RM). Following determination of 1RM outcomes, participants performed two sets of 10 repetitions with 2 min rest and recovery between sets, at the closest bar/leg press weight corresponding to 70% of familiarization session 1RM. Participants then rested for 2 min before performing a third set to failure. After 2 min of rest, participants followed the same procedure to determine leg press 1RM and leg press muscular endurance. Hand placement on the bench press bar and seat and foot positioning on the leg press were in the same position for all attempts and testing sessions. Test-to-test reliability of performing these tests in our lab on resistance-trained participants has yielded low C_v_s and high reliability for the bench press (1.9%, *r* = 0.94) and hip sled/leg press (0.7%, *r* = 0.91).

The initial strength tests were performed to pre-fatigue the participant before assessing recovery performance after supplement ingestion. The recovery muscular strength and endurance performance assessment involved performing a 1RM test and then one set to failure at 70% of the familiarization 1RM, following similar procedures as those described above. In this way, the effects of acute and short-term supplementation could be assessed on muscular strength and endurance recovery. Total 1RM weight lifted in kg and the number repetitions performed for each set using 70% of the familiarization weight (rounded to the nearest 2.27 kg or 5 Lbs. that could be put on the bar) were recorded. Total lifting volume was calculated by multiplying the 70% of 1RM weight lifted by the number of repetitions performed each set. Day-to-day test reliability for performing this performance test in our lab on resistance-trained participants has yielded a CV of 0.34 and an intraclass correlation coefficient of 0.99 for three sets of bench press total lifting volume and an intraclass correlation coefficient of 0.96 for three sets of leg press total lifting volume.

### 3.7. Cycling Time Trial Performance Assessment

Time trial performance was examined on a magnetically-braked cycle ergometer (Lode Sport Excalibur Sport 925900, Groningen, The Netherlands) over a distance of 4 km. The seat height, seat position, handlebar height, and handlebar position were recorded for each participant to use for each testing session. The test began with a 3-min warm-up, comprised of pedaling against a resistance of 25 W for the first minute, 50 W for the second minute, and 100 W for the third minute. At the completion of the warm up, a standardized resistance (4 J/kg/rev) was applied and the participant was instructed to complete the distance the shortest time possible. Upon completion, the participant was instructed to continue at a slow pace to facilitate recovery. Data were recorded as time to completion and average power in Watts. Mean test-retest reliability studies performed in our lab over repeated days revealed mean CVs for time to completion of 0.235, with a mean intraclass correlation of 0.850.

### 3.8. Statistical Analysis

Data were analyzed using IBM^®^ SPSS^®^ Version 24 software (IBM Corp., Armonk, NY, USA). The sample size was determined based on the expectation of a five percent improvement in exercise performance and corresponding power of 0.80 as well as prior research in our lab using a similar research design [61]. Baseline demographic data were analyzed using one-way ANOVA. Data were examined for a treatment order effect to confirm that randomization procedures were effective. Data were analyzed using univariate, multivariate and repeated measures general linear models (GLM), with and without gender as a covariate. Since results were consistent, results are reported without the covariate included. Wilks’ Lambda multivariate tests are reported to describe overall effects of related variables analyzed. Greenhouse–Geisser univariate tests with least significant difference post-hoc comparisons are presented for individual variables analyzed. Hematological variables were also examined relative to normal clinical limits, to examine the frequency of changes in hematology outside of normal, clinical limits, from baseline to follow-up, using Chi-square and adjusted residual analyses. These analyses examined the likelihood of excursions outside of clinical limits for each treatment, as follows: (1) no change; (2) normal at baseline, high at follow-up; (3) high at baseline, high at follow-up; (4) high at baseline, normal at follow-up. Delta changes (post–pre) were calculated on selected variables and analyzed by one-way ANOVA with Sidak post-hoc analyses. Data are reported as mean (SD), mean change from baseline (95% CI), and frequency of occurrence, according to the Chi-square analysis. Data were considered statistically significant when the probability of type I error was 0.05 or less, while tendencies towards statistical significance were noted when *p*-levels were *p* > 0.05 to *p* < 0.10. Partial eta squared effect sizes (η_p_^2^) were also reported on select variables as an indicator of effect size. An eta squared of around 0.02 was considered small, 0.13 medium, and 0.26 large. Primary outcomes included various indices of hematologic and hemodynamic changes accompanying supplementation. Secondary outcomes included various indices of exercise performance. Mean changes with 95% CIs completely above or below baseline were considered significantly different [62].

## 4. Results

### 4.1. Baseline Characteristics

A total of 38 individuals met the initial screening criteria and consented to participate in this study (Figure 3). Four participants declined to participate after undergoing familiarization assessments. Therefore, 34 individuals were allocated into treatments to begin the cross-over study. Six participants did not complete the entire study due to illness unrelated to the treatment: family emergency, and time constraints. Statistical analyses were performed on 28 individuals (18 men and 10) women (Table 1). No significant differences were observed among gender in age and body mass indices. However, as expected, men were taller, weighed more, had more fat free mass, and had less body fat than females.

### 4.2 Primary Outcome—Safety

#### 4.2.1. Hemodynamic Response

**Figure 4:**
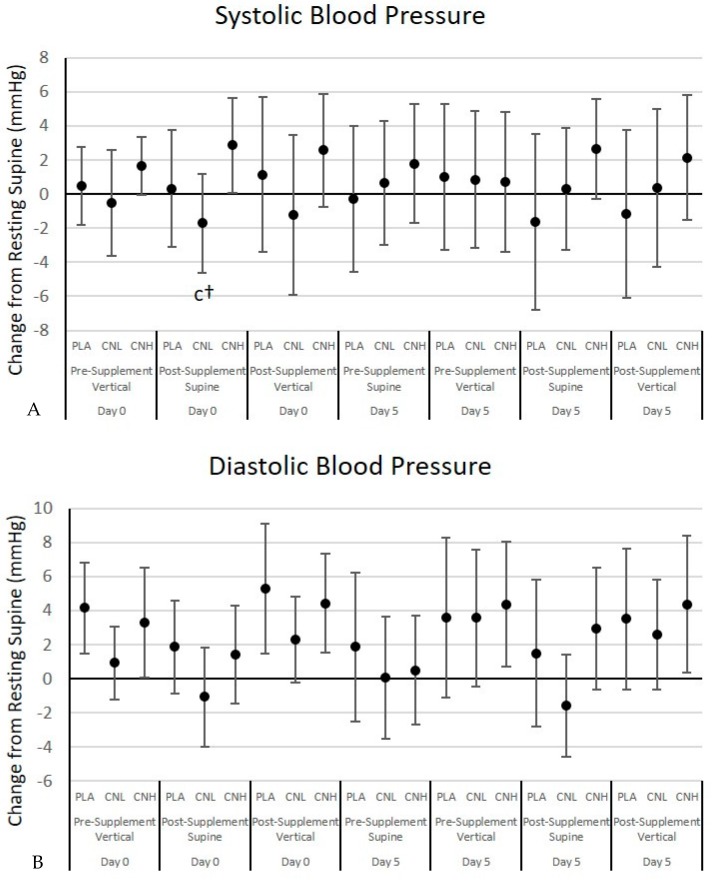

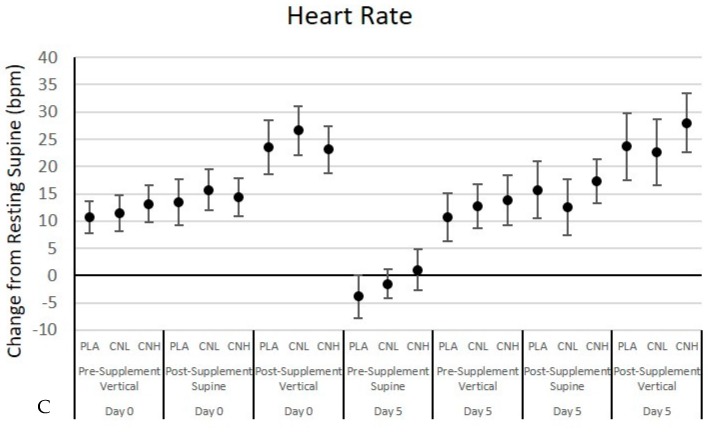
Systolic blood pressure (**A**), diastolic blood pressure (**B**), and heart rate (**C**) responses from to the postural hemodynamic challenge test from baseline values for the placebo (PLA), low dose creatine nitrate (CNL), and high dose creatine nitrate (CNH) treatments. Data are mean changes (95% CI). Confidence intervals not crossing zero are statistically significant (*p* < 0.05). c Represents *p* < 0.05 difference from CNH. † Represents *p* > 0.05 to *p* < 0.05 effect.

Table S1 presents data from the hemodynamic reactivity test. The multivariate analysis revealed no significant overall Wilks’ Lambda treatment × time (*p* = 0.38) or treatment × time × gender (*p* = 0.45) effects among (SBP), diastolic blood pressure (DBP), mean arterial pressure (MAP), pulse pressure (PP), heart rate (HR), or rate pulse product (RPP). Similarly, no significant univariate treatment × time or treatment × time × gender effects were observed for SBP, DBP, MAP, PP, HR, or RPP. Figure 4 shows mean changes with 95% CIs for SBP, DBP, and HR. No significant changes from baseline supine values were seen in SBP. Additionally, no significant (*p* < 0.05) differences were observed among treatments at any time point, although SBP, in the CNL treatment, tended to be lower than the CNH responses (*p* = 0.095). However, neither of these values differed from the PLA treatment (PLA: 1.1 [−3.4, 5.7], CNL: −1.2 [−5.9, 3.5], CNH: 2.5 [−0.8, 5.9] mmHg, *p* = 0.10). No significant changes were observed over time in DBP responses or among treatments at any data point. HR values significantly increased above pre-supplementation supine baseline values, as a result of moving to a vertical position and in response to exercise. However, no significant differences were observed among treatments at any data point.

#### 4.2.2. Hematology Assessment

Tables S2–S8 present the results of whole blood and serum markers monitored in this study. No significant overall multivariate or univariate treatment × time or treatment × gender × time interactions were observed. Table S9 shows Chi square analysis of changes from baseline values observed. No significant changes were observed among treatments. Overall, some perturbations in blood chemistries exceeding normal clinical limits at baseline and follow-up were observed before and after supplement ingestion. However, no significant differences were seen in the frequency of occurrences among treatments.

#### 4.2.3. Self-Reported Side Effects

Tables S10 and S11 present the frequency and severity of monitored side effects. Participants were asked to rate the frequency and severity of the following eight symptoms before and after testing each testing day: dizziness, headaches, tachycardia, heart palpitations, dyspnea, nervousness, blurred vision, and other symptoms. No significant differences at any time point among any of the treatments for frequency or severity of symptoms were found. Additionally, as Table S12 shows, creatine nitrate supplementation was not associated with dehydration.

### 4.3. Secondary Outcome—Performance

#### 4.3.1. Bench Press and Leg Press Performance

**Figure 5:**
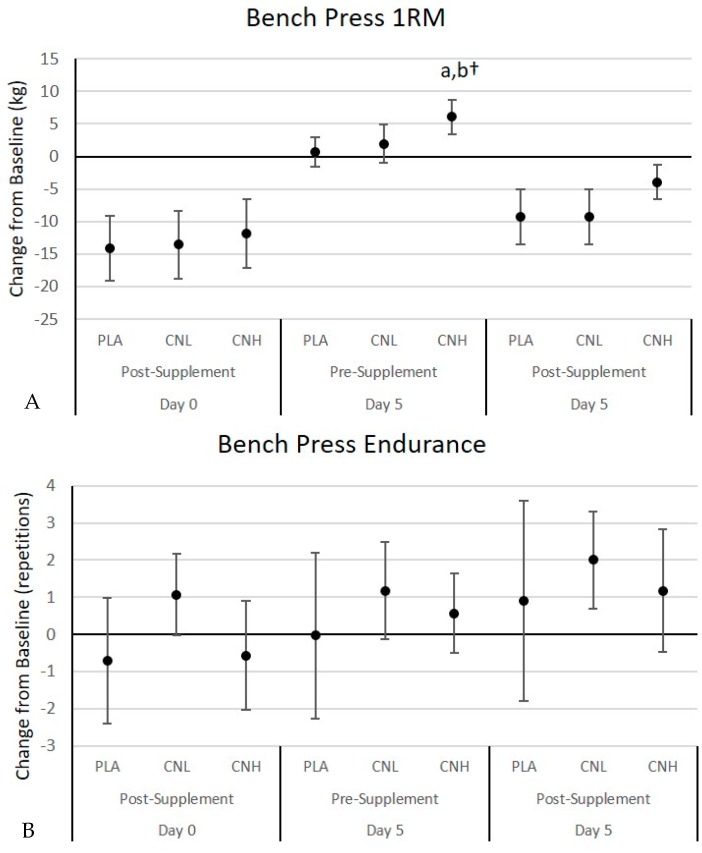

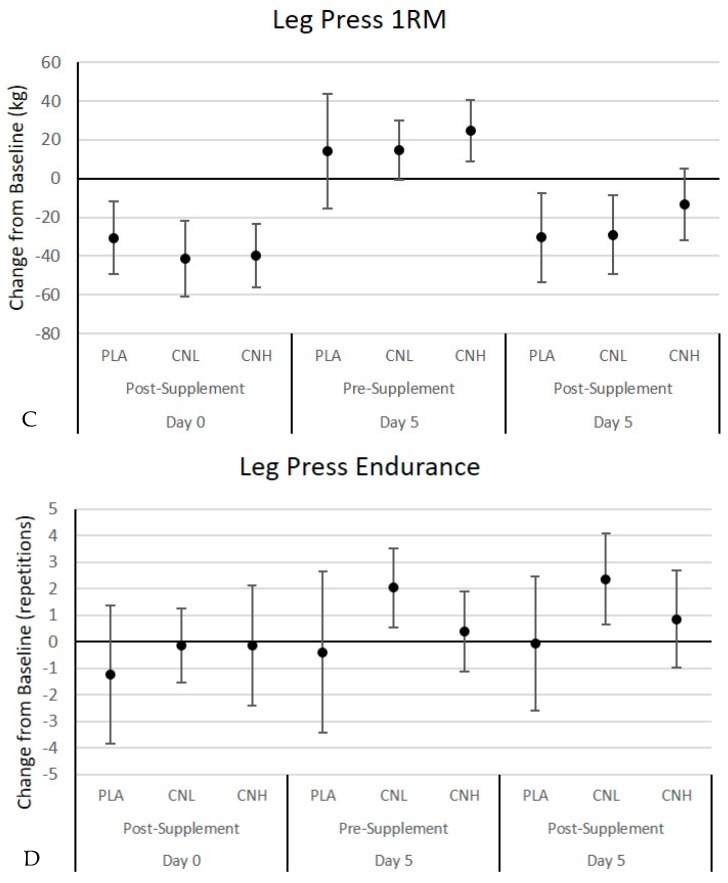
Bench press one repetition maximum (1 RM) (**A**), bench press endurance (**B**), leg press 1 RM (**C**) and leg press endurance (**D**) changes from baseline values for the placebo (PLA), low dose creatine nitrate (CNL), and high dose creatine nitrate (CNH) treatments. Data are mean changes (95% CI). Confidence intervals not crossing zero are statistically significant (*p* < 0.05). a Represents *p* < 0.05 difference from PLA. b Represents *p* < 0.05 difference from CNL. † Represents *p* > 0.05 to *p* < 0.05 effect.

Table 2 presents bench and leg press muscular strength and endurance performance results. Bench press and leg press 1RM results were analyzed in absolute (kg) and relative terms (kg/kg_FFM_) and since results were consistent, absolute values are reported. The multivariate analysis revealed overall Wilks’ Lambda treatment (*p* = 0.94), time (*p* = 0.001), gender (*p* = 0.001), treatment × time (*p* = 0.11), treatment × gender (*p* = 0.90), time × gender (*p* = 0.001), and treatment × time × gender (*p* = 0.57) effects. The univariate analysis revealed that although some time and gender effects were observed, no significant treatment × time or gender × time effects were seen in bench press 1RM, bench press endurance, leg press 1RM, or leg press endurance. A significant treatment × time × gender interaction was observed in leg press endurance (*p* = 0.04). Post-hoc analyses revealed that leg press endurance significantly increased from baseline measurements in the CNL female group after 5 days of supplementation, but these values were not different among treatments. Figure 5 presents the mean change from baseline with 95% CIs for these variables. Performing the initial 1RM test and three sets of 10 repetitions at 70% of 1RM, with the last set to failure, promoted fatigue, as expected. Therefore, recovery bench press and leg press 1RM significantly decreased in all treatments. After 5 days of supplementation, 1RM bench press performance was significantly higher than baseline values following CNH treatment (PLA: 0.7 [−1.6, 3.0], CNL: 2.0 [−0.9, 4.9], CNH: 6.1 [3.5, 8.7] kg, *p* = 0.01). Participants in the CNH treatment also tended to experience a lower degree of loss in 1RM strength during the recovery performance tests following supplementation, on day 5 (PLA: −9.3 [−13.5, −5.0], CNL: −9.3 [−13.5, −5.1], CNH: −3.9 [−6.6, −1.2] kg, *p* = 0.07). After 5 days, pre-supplementation 1RM leg press was significantly increased above baseline values, only with CNH treatment (PLA: 13.9 [−15.7, 43.5], CNL: 14.6 [−0.5, 29.7], CNH: 24.7 [8.8, 40.6] kg, *p* = 0.73) and participants were able to maintain strength during the recovery while experiencing a significant decrease in 1RM leg press strength from baseline values in the PLA and CNL treatments (PLA: −30.5 [−53.4, −7.7], CNL: −29.0 [−49.5, −8.4], CNH: −13.3 [−31.9, 5.3] kg, *p* = 0.42). In terms of muscular endurance, participants in the CNL treatment were able to perform more bench press repetitions at 70% of their 1RM during recovery after supplementation on day 5 (PLA: 0.4 [−0.8, 1.6], CNL: 0.9 [0.35, 1.5], CNH: 0.5 [−0.2, 0.3], *p* = 0.56), more leg press repetitions at 70% of 1RM prior to supplementation on day 5 (PLA: −0.2 [−1.6, 1.2], CNL: 0.9 [0.2, 1.6], CNH: 0.2 [−0.5, 0.9], *p* = 0.25) and after supplementation during recovery on day 5 (PLA: −0.03 [−1.2, 1.1], CNL: 1.1 [0.3, 1.9], CNH: 0.4 [−0.4, 1.2], *p* = 0.23).

#### 4.3.2. Cycling Time Trial Performance

**Figure 6:**
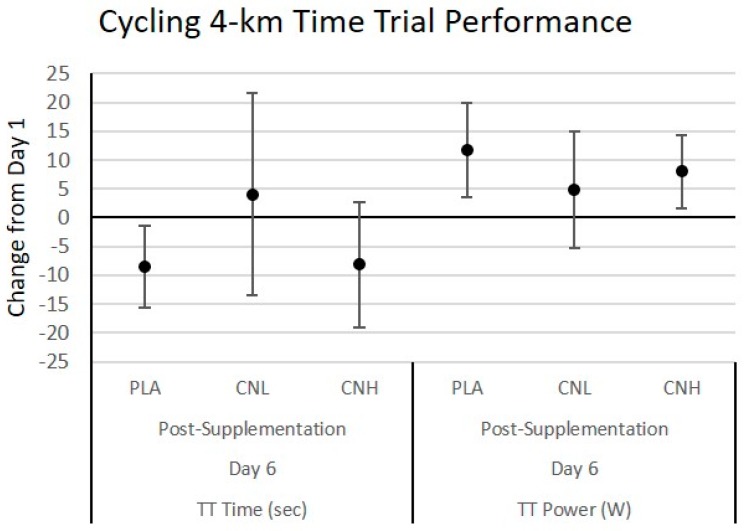
Change in 4 km cycling time trial performance from day 1 to day 6 for the placebo (PLA), low dose creatine nitrate (CNL), and high dose creatine nitrate (CNH) treatments. Data are mean changes (95% CI). Confidence intervals not crossing zero are statistically significant (*p* < 0.05).

Table 3 presents 4 km cycling time trial performance data. Power output data were analyzed in absolute (W) and relative terms (W/kg_FFM_) and since results were similar the absolute values are reported. The multivariate analysis revealed overall Wilks’ Lambda treatment (*p* = 0.79), time (*p* = 0.008), gender (*p* = 0.001), treatment × time (*p* = 0.20), treatment × gender (*p* = 0.85), time × gender (*p* = 0.22), and treatment × time × gender (*p* = 0.06) effects. The univariate analysis revealed significant gender (*p* = 0.001) and treatment × time × gender (*p* = 0.02) effects for time to completion and time (*p* = 0.005) and gender (*p* = 0.001) effects for mean power. The univariate analysis revealed a statistical trend in performance times (treatment × time effects *p* = 0.068, η_p_^2^ = 0.066) with a significant treatment × time × gender interaction (*p* = 0.02). Post-hoc analyses revealed that females in the CNL treatment performed the time trial slower (392 ± 75 to 416 ± 114 s) compared to females in the CNH treatment, who saw improved performance (402 ± 80 to 381 ± 85 s). These values were significantly different than day 1 performance as well as significantly different than male times. Figure 6 presents the change in 4 km time trial time performance (seconds) and average power output observed during the time trials. Participants experienced significant improvements in performance times from baseline following PLA treatment, while average power output was improved over baseline values in the PLA and CNH treatments. Improvements in performance times were nearly identical in the CNH treatment, but were not significant, due to a larger 95% CI range observed.

## 5. Discussion and Conclusions

Creatine and nitrates are popular dietary supplements for active individuals, but little is known regarding the effects of co-ingestion relative to safety and/or performance. Only two previous studies [51,52] have examined creatine nitrate alone and two other studies [53] have examined creatine nitrate as a part of a multiple ingredient supplement. Each of these studies found that creatine nitrate appears to be safe for the doses (1–3 g/day) and duration (up to 8 weeks) examined. The current study examined the effects of ingesting 3 g and 6 g doses of creatine nitrate over a 6-day period, prior to and/or following intense exercise on hemodynamic responses to a postural challenge, fasting blood clinical health markers, self-reported side effects, and exercise performance and recovery. Results indicate that acute and short-term creatine nitrate supplementation appears safe and may provide some ergogenic benefit to resistance exercise and recovery.

### 5.1. Primary Outcome—Safety

As expected, significant time effects were seen for resting blood pressure and heart rate, in response to moving from a supine to vertical position as well as in response to exercise. Previous research has shown little change in systolic blood pressure, but an increase in diastolic blood pressure and heart rate when comparing supine to standing positions at rest [63]. Heart rate and blood pressure are generally higher when exercising in the upright versus supine position, in order to enhance venous return [64]. Our findings support these findings in that we observed changes in heart rate and blood pressure in response to changes in posture and exercise.

Prior nitrate supplementation studies have reported decreases in blood pressure after supplementation [25,65,66,67,68]. For example, Siervo and colleagues [69] conducted a meta-analysis on 16 crossover nitrate studies, that had durations ranging from 2 h to 15 days, and washout periods ranging from 6 to 28 days. The researchers reported that beetroot juice or inorganic nitrate ingestion was associated with a decrease in SBP (−4.4 [−5.9, −2.8] mmHg, *p* < 0.001) but not DBP (−1.1 [−2.2, 0.1] mmHg, *p* = 0.06). The dose of nitrates ranged from ~150 mg to ~3 g per day. Six studies (38%) found no change in SBP and nine (56%) found no change in DBP. Moreover, neither the duration of supplementation, nor source of nitrate were found to be associated with decreased blood pressure. However, a significant correlation (*p* < 0.05) was found between the dosage of nitrate and reduction in SBP.

Studies examining the effects of ingesting creatine nitrate independently [51,52], or as part of a multi-ingredient supplement [53,54,70], have not found similar results. Our study is in agreement with these studies, as although time effects were seen as a result of changing body position and in response to exercise, no treatment × time interaction effects were observed in resting or post-exercise SBP, DBP, MAP, PP, HR, or RPP. While dietary nitrates may help individuals manage blood pressure, adding additional nitrates through creatine nitrate (up to 2 g per dose and 4 g/day for 7 days), on top of those supplied by the diet (i.e., 0.12–1.2 g per day) does not appear to produce a significant reduction in resting blood pressure [52]. These data refute concerns that creatine nitrate ingestion (up to 6 g per dose) would pose a risk of hypotension at rest or following strenuous exercise.

Our study also found no significant differences among treatments for fasting blood clinical markers. Additionally, we did not observe a significant increase in the number of normal clinical values changing to above normal clinical values for any of the blood chemistry parameters measured. Likewise, no significant differences were seen among treatments in self-reported frequency or severity of side effects. These findings are in agreement with other creatine nitrate studies [51,52,53]. Collectively, current and prior research findings support contentions that creatine nitrate supplementation is safe when apparently healthy men and women ingest creatine nitrate in doses of up to 6 g or 12 g/day for a week and 3 g/day for up to 8 weeks [31,51,52,53,54].

### 5.2. Secondary Outcome—Performance

The current study allowed for the assessment of acute and short-term effects of creatine nitrate on performance as well as recovery from intense resistance exercise. While significant time effects were found for the performance variables, no significant treatment or treatment × time interactions were found. However, analyses of mean changes from baseline with 95% CIs revealed some ergogenic benefit. In this regard, significant improvements in bench press and leg press 1RM as well as leg press endurance were observed after 5 days of supplementation with creatine nitrate ingestion. Further, there was evidence that recovery 1RM strength and muscular endurance was better maintained after creatine nitrate ingestion on day 5. Benefits were seen with both 3 g and 6 g doses. These findings support previous reports that creatine nitrate may provide some ergogenic benefit to high intensity intermittent exercise capacity and/or recovery [52,53]. Since we previously reported that ingestion of 6 g/day of creatine nitrate for 7 days did not affect muscle creatine levels [52], the ergogenic benefit observed is most likely be due to the nitrate ingestion. Present findings are in agreement with a number of studies that indicate that nitrate supplementation can affect high intensity intermittent exercise capacity [32,36,37,71,72,73]. Interestingly though, in contrast to other reports [27,74], we did not observe an improvement in 4 km cycling time trial performance. The most likely explanation is that while these participants were accustomed to resistance training, they were not trained cyclists or familiar with performing cycling time trials. Consequently, variability would be expected to be higher on the cycling time trial, which may have influenced results.

### 5.3. Limitations

Our study has several limitations that should be noted. First, since we previously reported that 7 days of supplementation with 6 g/day of creatine nitrate had no effects on muscle creatine levels [52], we did not feel obtaining muscle biopsies to determine muscle creatine and phosphocreatine content was justified in this study. Thus, it is possible that increases in muscle creatine or phosphocreatine contributed to performance outcomes. It is also possible that while participants were instructed to maintain their normal diet, variations in dietary intake of creatine and/or nitrates may have affected responsiveness to creatine nitrate supplementation. Second, our study was relatively short in duration. While prior research has shown that acute and short-term nitrate supplementation can affect high intensity intermittent exercise, greater benefits from creatine supplementation would likely take higher short-term daily doses of creatine (e.g., 0.3 g/kg/day or 20 g/day) to observe ergogenic benefits. However, since creatine nitrate is relatively new to the market place and we examined higher than the recommended acute doses (i.e., 3 and 6 g per serving, compared to 1–2 g per serving found in most supplements), we felt that this protocol was a good initial evaluation of the safety and potential ergogenic benefits of higher doses of creatine nitrate. Finally, we examined 4 km cycling time trial performance in resistance trained athletes rather than trained cyclists. It is possible that greater ergogenic benefits may be observed in athletes accustomed to performing cycling time trials.

### 5.4. Conclusion

Acute and short-term creatine nitrate supplementation (3 g and 6 g doses) did not alter resting or post exercise responses to a hemodynamic challenge, significantly alter fasting clinical blood profiles, or increase the frequency or severity of self-reported side effects. Therefore, creatine nitrate supplementation appears to be safe when administered to healthy men and women within the context of this study. Short-term creatine nitrate supplementation enhanced 1RM bench press, 1RM leg press, and leg press muscular endurance. It also helped participants maintain 1RM strength and/or muscular endurance capacity to a better degree after performing exhaustive resistance exercise. However, creatine nitrate supplementation had no effect on 4 km cycling time trial performance in non-cycling trained athletes. Future research should evaluate the effects of varying doses and lengths of supplementation of creatine nitrate on different types of exercise performance, whether creatine nitrate may affect the responsiveness to creatine loading, and/or how ingestion of creatine nitrate impacts on creatine transport, creatine retention, and protein synthesis. Additionally, more research should be conducted to evaluate the effects of co-ingesting creatine nitrate with effective doses of creatine monohydrate and/or the effects of ingesting creatine monohydrate with other forms of nitrates on exercise capacity; and, finally, given the potential health benefits noted with creatine and nitrates, research should also explore potential synergistic clinical applications and mechanisms of action.

## Figures and Tables

**Table 1:** Participant Characteristics.

	Total	Male	Female	*p*-Level
*N*	28	18	10	
Age (years)	21.6 ± 3.7	21.4 ± 3.0	22.1 ± 4.7	0.40
Height (m)	1.72 ± 0.08	1.76 ± 0.06	1.65 ± 0.06 ^†^	0.001
Weight (kg)	73.1 ± 11.4	76.6 ± 9.0	66.9 ± 12.6 ^†^	0.001
Body Mass Index (BMI) (kg/m^2^)	24.7 ± 2.8	24.8 ± 2.9	24.5 ± 2.8	0.63
Fat Free Mass (kg)	53.5 ± 10.3	59.9 ± 6.1	41.9 ± 4.2 ^†^	0.001
Fat Mass (kg)	13.8 ± 8.0	10.7 ± 6.2	19.6 ± 7.7 ^†^	0.001
Body Fat (%)	20.4 ± 10.5	14.6 ± 7.0	30.9 ± 7.1 ^†^	0.001

Data are mean ± SD. *p* < 0.05 is considered significant. (^†^) denotes a significant difference from male.

**Table 2:** Bench Press and Leg Press Performance.

Variable	Treatment	Day				Mean	Interaction	*p*-Level
		0 Pre	0 Post	5 Pre	5 Post			
Bench Press	Overall	73.9 ± 30.0	67.9 ± 28.4 *	75.2 ± 30.4 *	70.5 ± 28.9 *	71.9 ± 29.4	Time	0.001
1RM (kg)	PLA	74.4 ± 30.7	68.1 ± 28.3	74.8 ± 30.4	70.3 ± 29.2	72.5 ± 29.1	Treatment	0.94
	CNL	73.1 ± 29.7	67.1 ± 28.3	74.1 ± 30.2	69.0 ± 28.7	70.4 ± 28.8	Treatment × Time	0.46
	CNH	74.1 ± 30.9	68.8 ± 29.7	77.0 ± 31.6	72.4 ± 30.1	73.0 ± 30.7		
	Male	92.4 ± 19.9	84.4 ± 21.2 *	93.7 ± 20.5 *	87.6 ± 21.1 *	89.3 ± 21.0	Gender	0.001
	Female	40.5 ± 7.7 ^†^	38.2 ± 8.4 ^†,^*	41.8 ± 8.8 ^†,^*	39.6 ± 7.6 ^†^	40.0 ± 8.6 ^†^	Time × Gender	0.001
	PLA M	92.8 ± 21.1	84.0 ± 21.6	92.8 ± 21.2	86.9 ± 22.1	88.9 ± 21.2	Treatment × Gender	0.95
	PLA F	41.3 ± 9.4	39.3 ± 9.1	42.2 ± 9.9	40.2 ± 8.2	41.0 ± 9.3	T × T × G	0.96
	CNL M	91.4 ± 19.7	83.4 ± 21.1	92.2 ± 20.6	85.8 ± 20.8	87.3 ± 20.8		
	CNL F	40.2 ± 6.8	37.5 ± 7.5	41.3 ± 8.6	38.6 ± 7.1	39.4 ± 7.8		
	CNH M	93.1 ± 20.0	85.9 ± 22.1	96.2 ± 20.8	90.2 ± 21.2	91.7 ± 21.2		
	CNH F	40.5 ± 7.7	37.9 ± 9.2	42.0 ± 8.9	40.2 ± 8.1	39.7 ± 8.6		
Bench Press	Overall	14.1 ± 5.3	14.1 ± 4.7	14.7 ± 5.0	15.5 ± 5.3 *	14.3 ± 5.1	Time	0.006
Endurance	PLA	14.8 ± 5.9	14.0 ± 5.0	14.7 ± 5.6	15.6 ± 6.3	14.8 ± 5.8	Treatment	0.55
(Repetitions)	CNL	12.9 ± 4.0	14.0 ± 4.4	14.1 ± 4.6	14.9 ± 4.5	13.5 ± 4.1	Treatment × Time	0.76
	CNH	14.8 ± 5.6	14.2 ± 4.9	15.3 ± 4.9	15.9 ± 4.9	14.7 ± 5.2		
	Male	13.7 ± 4.8	13.2 ± 4.7	14.1 ± 4.9	14.5 ± 5.2	13.5 ± 4.9	Gender	0.04
	Female	14.9 ± 6.0	15.6 ± 4.4	15.9 ± 5.2	17.2 ± 4.9	15.8 ± 5.3 ^†^	Time × Gender	0.34
	PLA M	13.4 ± 4.6	12.7 ± 4.7	12.9 ± 3.9	14.0 ± 5.2	13.0 ± 4.5	Treatment × Gender	0.26
	PLA F	17.2 ± 7.4	16.4 ± 5.0	18.0 ± 6.8	18.6 ± 7.2	18.3 ± 6.5	T × T × G	0.62
	CNL M	12.6 ± 4.4	13.3 ± 5.0	13.7 ± 5.3	14.3 ± 5.2	13.0 ± 4.7		
	CNL F	13.4 ± 3.3	15.1 ± 3.3	14.7 ± 3.1	16.0 ± 2.6	14.2 ± 2.7		
	CNH M	15.2 ± 5.3	13.5 ± 4.8	15.6 ± 5.1	15.3 ± 5.5	14.6 ± 5.3		
	CNH F	14.0 ± 6.3	15.4 ± 5.1	14.9 ± 4.8	17.1 ± 3.7	14.8 ± 5.0		
Leg Press	Overall	408 ± 123	391 ± 121 *	417 ± 124 *	397 ± 122 *	403 ± 119	Time	0.001
1RM (kg)	PLA	411 ± 122	397 ± 119	417 ± 125	397 ± 117	407 ± 115	Treatment	0.66
	CNL	397 ± 122	379 ± 119	404 ± 122	384 ± 120	387 ± 114	Treatment × Time	0.62
	CNH	417 ± 129	399 ± 128	428 ± 127	411 ± 132	414 ± 127		
	Male	476 ± 96	456 ± 98	483 ± 98	464 ± 97	466 ± 94	Gender	0.001
	Female	286 ± 51	276 ± 50	297 ± 55	278 ± 48	287 ± 54 ^†^	Time × Gender	0.38
	PLA M	474 ± 99	457 ± 99	479 ± 105	461 ± 92	464 ± 95	Treatment × Gender	0.88
	PLA F	296 ± 59	288 ± 61	305 ± 63	282 ± 46	299 ± 58	T × T × G	0.97
	CNL M	463 ± 95	443 ± 94	470 ± 95	449 ± 96	448 ± 90		
	CNL F	278 ± 53	262 ± 44	285 ± 53	268 ± 50	275 ± 52		
	CNH M	491 ± 96	467 ± 104	500 ± 96	482 ± 106	485 ± 96		
	CNH F	284 ± 45	276 ± 47	300 ± 52	283 ± 52	286 ± 50		
Leg Press	Overall	20.8 ± 7.7	20.3 ± 8.1	21.5 ± 7.7	21.8 ± 7.4	21.3 ± 7.7	Time	0.06
Endurance	PLA	21.8 ± 9.3	20.5 ± 8.7	21.4 ± 8.2	21.7 ± 8.2	21.8 ± 8.5	Treatment	0.7
(Repetitions)	CNL	19.0 ± 7.3	18.9 ± 7.8	21.1 ± 8.3	21.4 ± 7.2	20.2 ± 7.8	Treatment × Time	0.23
	CNH	21.6 ± 5.9	21.4 ± 7.9	22.0 ± 6.7	22.4 ± 6.7	21.9 ± 6.7		
	Male	21.6 ± 7.2	20.6 ± 6.3	21.8 ± 6.9	22.5 ± 6.5	21.4 ± 6.9	Gender	0.35
	Female	19.3 ± 8.3	19.7 ± 10.6	20.9 ± 9.0	20.6 ± 8.6	21.2 ± 9.1	Time × Gender	0.56
	PLA M	21.7 ± 9.1	20.2 ± 6.1	20.8 ± 6.4	22.6 ± 7.3	21.2 ± 7.5	Treatment × Gender	0.72
	PLA F	21.9 ± 10.3	21.0 ± 12.4	22.3 ± 11.1	20.1 ± 10.0	22.9 ± 10.2	T × T × G	0.04
	CNL M	19.9 ± 6.5	19.9 ± 6.3	20.7 ± 7.6	21.8 ± 5.4	20.3 ± 6.6		
	CNL F	17.5 ± 8.7	17.0 ± 9.9	21.8 ± 9.7 *	20.7 ± 10.1	20.1 ± 9.8		
	CNH M	23.3 ± 5.7	21.6 ± 6.8	23.9 ± 6.4	23.2 ± 7.1	22.7 ± 6.6		
	CNH F	18.5 ± 5.4	21.1 ± 10.0	18.5 ± 6.0	21.1 ± 6.1	20.6 ± 6.8		

Data are means ± SD. The multivariate analysis revealed overall Wilks’ Lambda values for treatment (*p* = 0.94), time (*p* = 0.001), gender (*p* = 0.001), treatment × time (*p* = 0.11), treatment × gender (*p* = 0.90), time × gender (*p* = 0.001), and treatment × time × gender (*p* = 0.57). Greenhouse–Geisser univariate p-levels are presented for each variable. PLA = placebo (0 g), CNL = creatine nitrate low dose (3 g), CNH = creatine nitrate high dose (6 g), M = male, F = female, 1RM = one repetition maximum, and T × T × G = time × treatment × gender interaction. *p* < 0.05 is considered significant. Statistical notations. (*) Denote a significant difference from baseline. (^†^) Denotes a significant difference from male.

**Table 3:** Cycling Time Trial Performance.

	Treatment	Day		Mean	Interaction	*p*-Level
		1	6			
Time	Overall	275 ± 103	270 ± 110	272 ± 106	Time	0.34
(seconds)	PLA	271 ± 100	263 ± 105	267 ± 102	Treatment	0.45
	CNL	282 ± 99	286 ± 122	284 ± 110	Treatment × Time	0.068
	CNH	271 ± 113	262 ± 105	267 ± 108		
	Male	210 ± 35	204 ± 36	207 ± 35	Gender	0.00
	Female	391 ± 79	390 ± 98	390 ± 88 ^†^	Time × Gender	0.47
	PLA M	212 ± 35	201 ± 36	207 ± 35	Treatment × Gender	0.73
	PLA F	378 ± 89 ^†^	374 ± 98 ^†^	376 ± 91	T × T × G	0.02
	CNL M	220 ± 37	214 ± 39	217 ± 38		
	CNL F	392 ± 75 ^†^	416 ± 114 ^†,^*	404 ± 95		
	CNH M	198 ± 32	197 ± 31	197 ± 31		
	CNH F	402 ± 80 ^†^	381 ± 85 ^†,^*	392 ± 81		
Mean Power	Overall	245 ± 80	253 ± 86 *	249 ± 83	Time	0.005
(W)	PLA	246 ± 79	258 ± 86	252 ± 82	Treatment	0.55
	CNL	237 ± 74	242 ± 85	240 ± 79	Treatment × Time	0.47
	CNH	252 ± 88	260 ± 87	256 ± 87		
	Male	293 ± 55	304 ± 59	299 ± 57	Gender	0.00
	Female	159 ± 27	162 ± 32	160 ± 29 ^†^	Time × Gender	0.10
	PLA M	291 ± 57	308 ± 62	300 ± 59	Treatment × Gender	0.72
	PLA F	165 ± 31	168 ± 33	167 ± 31	T × T × G	0.30
	CNL M	282 ± 49	291 ± 61	286 ± 55		
	CNL F	157 ± 26	153 ± 35	155 ± 30		
	CNH M	306 ± 57	313 ± 56	310 ± 56		
	CNH F	154 ± 25	163 ± 30	159 ± 27		

Data are means ± SD. The multivariate analysis revealed overall Wilks’ Lambda treatment (*p* = 0.79), time (*p* = 0.008), gender (*p* = 0.001), treatment × time (*p* = 0.20), treatment × gender (*p* = 0.85), time × gender (*p* = 0.22), and treatment × time × gender (*p* = 0.06) effects. Greenhouse–Geisser univariate *p*-levels are presented for each variable. PLA = placebo treatment, CNL = creatine nitrate low dose treatment, CNH = creatine nitrate high dose treatment, M = male, F = female, and T × T × G = time × treatment × gender interaction. *p* < 0.05 is considered significant. Statistical notations. (*) Denote a significant difference from baseline. (^†^) Denotes a significant difference from male.

## Supplementary Materials

The following are available online at www.mdpi.com/2072-6643/9/12/1359/s1, Table S1: Hemodynamic Response, Table S2: White Blood Cell Counts, Table S3: Red Blood Cell Counts, Table S4: Kidney Function Markers, Table S5: Liver Enzymes, Table S6: Muscle Catabolism Markers, Table S7: Lipid Profile, Table S8: Blood Glucose, Table S9: Assessment of blood chemistry changes from baseline to day 6, Table S10: Frequency of Side Effects, Table S11: Severity of Side Effects, Table S12: Body Water Data.
